# Treatment of Patients With Extraskeletal Ewing Sarcoma in the Scapular and Arm Regions With Wide Resection: A Report of Two Cases and Literature Review

**DOI:** 10.7759/cureus.75848

**Published:** 2024-12-17

**Authors:** Arın Celayir, Yahya Deniz, Mete Ozer, Mahmut K Ozsahin, Huseyin Botanlioglu

**Affiliations:** 1 Department of Orthopaedics and Traumatology, Cerrahpasa Faculty of Medicine, Istanbul University - Cerrahpasa, Istanbul, TUR

**Keywords:** chemotherapy, extraskeletal ewing sarcoma, pnet, small round cell sarcomas, wide resection

## Abstract

Extraskeletal Ewing sarcoma (EES) is a rare and aggressive malignancy originating in soft tissues, distinct from osseous Ewing sarcoma. It commonly affects adolescents and young adults but can occur at any age. Due to its rarity and overlapping clinical features with other malignancies, EES poses significant diagnostic and therapeutic challenges. Accurate diagnosis requires a multidisciplinary approach, incorporating imaging, histopathological evaluation, and molecular testing, such as the detection of the EWSR1-FLI1 fusion protein. This case series highlights two instances of EES involving the scapular and arm regions. The clinical presentations, diagnostic workup, and management strategies, including surgery, chemotherapy, and radiotherapy, are detailed. These cases underscore the importance of a collaborative and individualized approach to managing EES. They also contribute valuable insights to the understanding and treatment of this rare malignancy, emphasizing the need for ongoing research and multidisciplinary collaboration in achieving optimal outcomes.

## Introduction

Extraskeletal Ewing sarcoma (EES) is a rare and aggressive malignant tumor originating in soft tissues, distinct from the more common osseous Ewing sarcoma [1]. It constitutes only 6-7% of all bone and soft tissue tumors. Unlike osseous Ewing sarcoma, which primarily affects younger individuals aged 5-25 years, EES predominantly occurs in adult males [2]. Common sites of involvement include the paravertebral region and retroperitoneum, with extremity involvement being rare. The prognosis of EES remains guarded, with a reported 10-year survival rate of approximately 55-60% when managed with a combination of chemotherapy, radiotherapy, and surgical resection [3].

Clinically, EES typically presents with localized pain and swelling. Imaging studies such as magnetic resonance imaging (MRI) are crucial for assessing tumor extent, while PET scans aid in detecting metastatic disease. The "onion-skin" periosteal reaction, commonly associated with osseous Ewing sarcoma, may also be observed in some cases of EES [4]. Histologically, EES is characterized by sheets of small, round, undifferentiated cells with aggressive growth and a high metastatic potential, particularly to the lungs, lymph nodes, skeleton, and brain [5]. Molecularly, the EWSR1-FLI1 fusion protein is a diagnostic hallmark frequently identified in patients with EES [6].

The 2020 World Health Organization (WHO) classification of tumors of soft tissue and bone introduced a dedicated chapter for undifferentiated small round cell sarcomas, encompassing Ewing sarcoma and three newly recognized subtypes: round cell sarcomas with EWSR1:non-ETS fusions, *CIC*-rearranged sarcoma, and sarcomas with *BCOR* genetic alterations [7]. EES remains a rare and distinct entity within this classification, with unique clinical and pathological features.

Treatment of EES requires a multidisciplinary approach, combining surgical resection, chemotherapy, and radiotherapy. While the overall survival rate for osseous Ewing sarcoma can reach 70%, particularly in pediatric populations, the prognosis for EES is slightly less favorable. Prognostic factors include tumor size, blood markers, metastatic status, and the ability to achieve complete surgical resection [8,9].

In this case report, we present two patients with EES involving the scapular and arm regions who were evaluated and treated at our clinic. Both patients presented with complaints of shoulder pain. Comprehensive treatment plans were developed by a multidisciplinary sarcoma team, and informed consent was obtained prior to the initiation of any procedures. Herein, we discuss their surgical management and postoperative outcomes.

## Case presentation

Case 1

**Figure 1:**
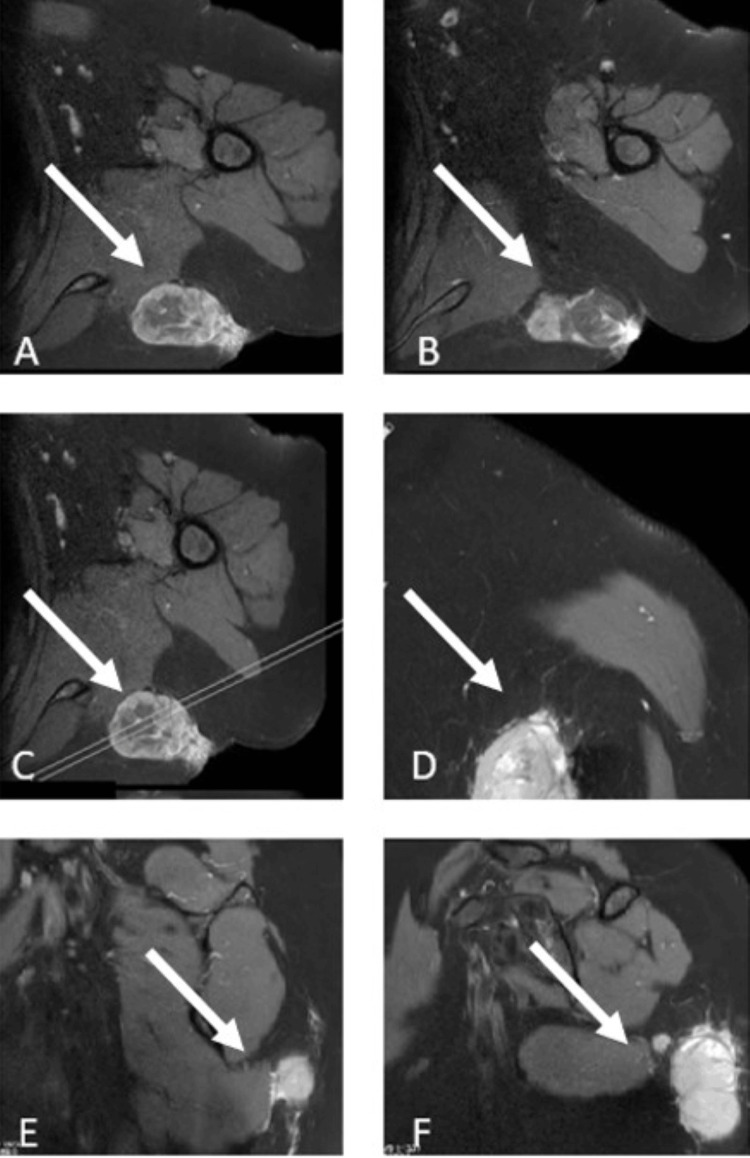
The preoperative magnetic resonance imaging (MRI) of the first patient, a 60-year-old female, demonstrates a lesion within the soft tissue of the left scapular region. The white arrows indicate the mass in the interscapular region.

The first patient was a 60-year-old woman who presented with severe shoulder pain and a noticeable mass in the left scapular region. The patient reported first noticing the mass five years prior, with progressive enlargement over time. In 2021, an open biopsy performed at an external center identified the mass as a "high-grade" round cell malignant tumor consistent with Ewing sarcoma. On examination, her shoulder range of motion was preserved and within normal limits. Preoperative assessments revealed a lesion in the soft tissue around the left interscapular region, measuring 53 × 43 × 25 mm (Figure 1).

**Figure 2:**
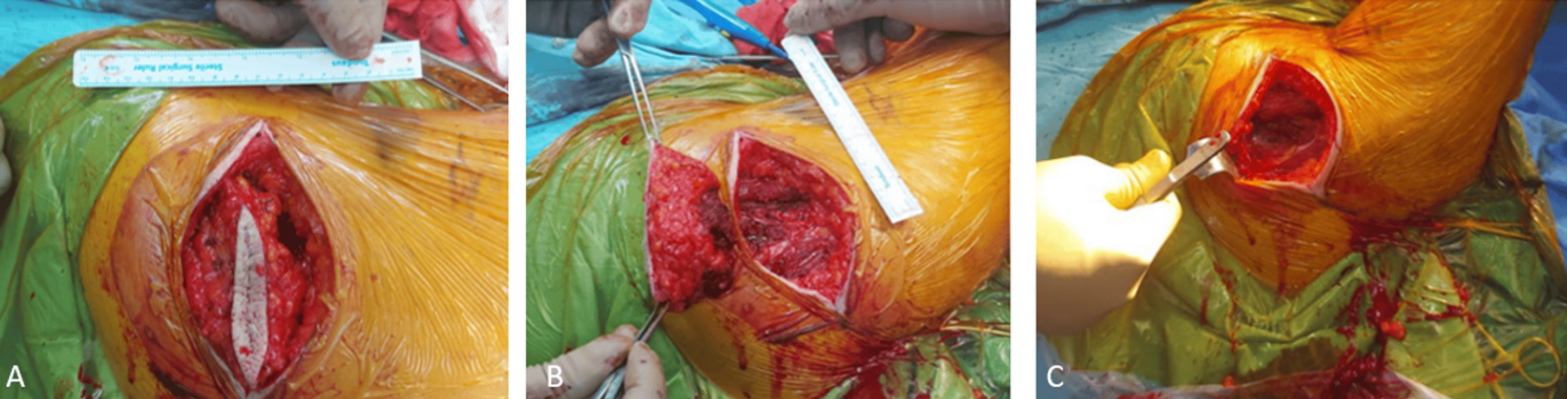
Intraoperative images of the first patient showing the lesion removed through wide resection, including the previous biopsy tract. (A) Image of the lesion before removal, (B) image during the removal of the lesion, and (C) image after the lesion was removed.

A wide surgical resection of the lesion was performed, ensuring the inclusion of the previous biopsy tract. The resected lesion measured 10 × 8 × 6 cm in size (Figure 2).

Postoperatively, the surgical sites showed no signs of discharge, and the patient was discharged within the first week. Follow-up appointments were scheduled regularly at the outpatient clinic. After discharge, the patient was referred to medical and radiation oncology for further evaluation, as per the decision of the multidisciplinary tumor board. It was determined that the patient would undergo both chemotherapy and radiotherapy in the postoperative period.

The chemotherapy regimen was administered in three phases. Initially, the patient received a combination of doxorubicin, ifosfamide, vincristine, and mesna. This was followed by a regimen comprising cyclophosphamide, mesna, and etoposide. Finally, she was treated with a regimen including doxorubicin, ifosfamide, mesna, and vincristine. Additionally, the patient underwent two courses of postoperative radiotherapy.

During the 1.5-year follow-up period, the patient showed no evidence of recurrence or complications at the surgical sites, demonstrating a favorable postoperative outcome.

Case 2

**Figure 3:**
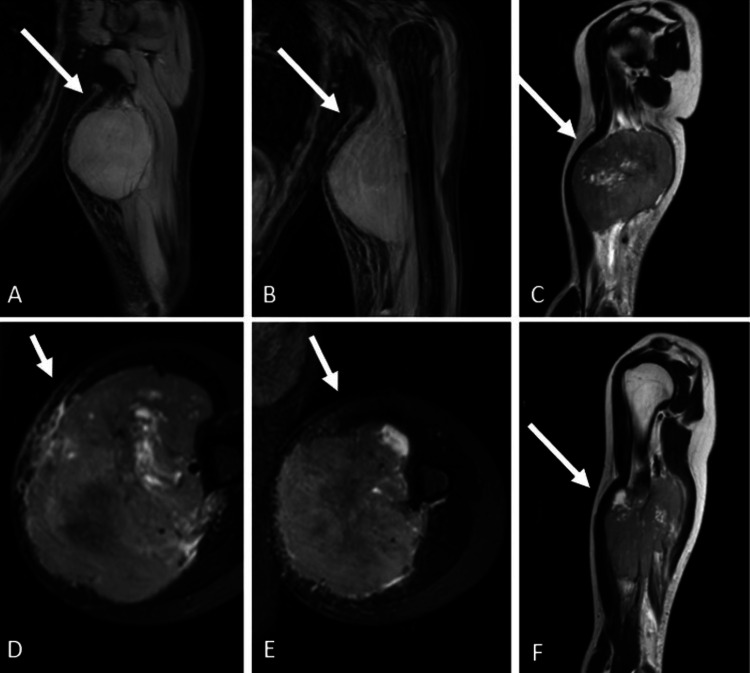
Preoperative magnetic resonance imaging (MRI) of the second patient, an 18-year-old female, showing the lesion located in the left shoulder region, surrounding the biceps brachii muscle: (A,B,C,F) sagittal and (D,E) axial MRI sections. The white arrows indicate the lesion surrounding the biceps, triceps, and brachioradialis muscles.

An 18-year-old female presented with left arm pain, which she had initially experienced two years earlier. During the second trimester of her pregnancy in 2021, she noticed swelling and a mass in her arm. The patient sought medical attention at an external medical center, where a mass measuring 61 × 27 mm was evaluated on March 25, 2022. Subsequently, she presented to our hospital for further evaluation and management (Figure 3).

On June 30, 2022, an MRI revealed a soft tissue mass measuring 72 × 86 × 102 mm. The mass displayed T1 hypointensity and T2 heterogeneous hyperintensity, located near the mid-segment of the humerus, between the planes of the biceps and triceps long head muscles. The mass was surrounded by the brachial neurovascular bundle and was in close proximity to the bone cortex, although the precise relationship with the cortex could not be clearly determined. Additionally, the MRI indicated widespread fusiform edema in the surrounding subcutaneous soft tissues.

On July 21, 2022, a core needle biopsy of the left arm was performed in the interventional radiology department of our hospital. The biopsy confirmed the diagnosis of Ewing sarcoma.

A 7 cm mass extending from the medial aspect of the left arm to the axilla was marked for surgical intervention, and a 15 cm incision was made. Dissection reached the biceps brachii muscle, which was extensively resected during the procedure. The brachial vein and artery were identified and ligated.

Tissue samples were sent for frozen section analysis, all of which confirmed negative surgical margins. The dissection was extended to the lateral aspect of the biceps brachii muscle, and the lateral segment was excised with careful control of collateral vessels. Posterior-medial dissection provided access to the triceps brachii muscle, which was released from the posterior-medial side with ligation of associated vascular and nerve structures.

The resected tissue was elevated as a flap, and further dissection of the brachial muscle and vascular-nerve structures from the posterior aspect of the tissue was performed. Extensive adhesions were noted between the tumoral tissue, surrounding muscles, vascular-nerve structures, and vascular adventitia. The tumor was carefully separated from the posterior vascular-nerve structures. During the procedure, an axillary vein injury occurred and was successfully repaired by a cardiovascular surgeon.

**Figure 4:**
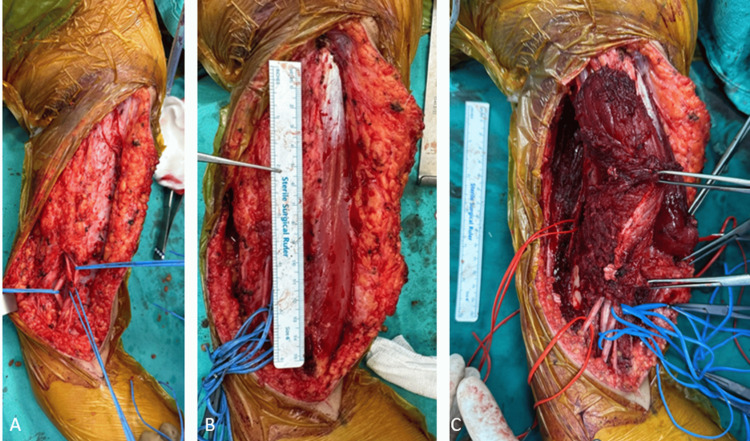
Intraoperative images of the second patient, showing the lesion with invasion involving the brachialis, biceps brachii, and triceps brachii muscles. (A) Images showing the vascular structures carefully separated from the surrounding muscle tissue, (B) a pre-resection image of the mass, and (C) the preservation of vascular structures during the resection of the biceps and brachialis muscles.

**Figure 5:**
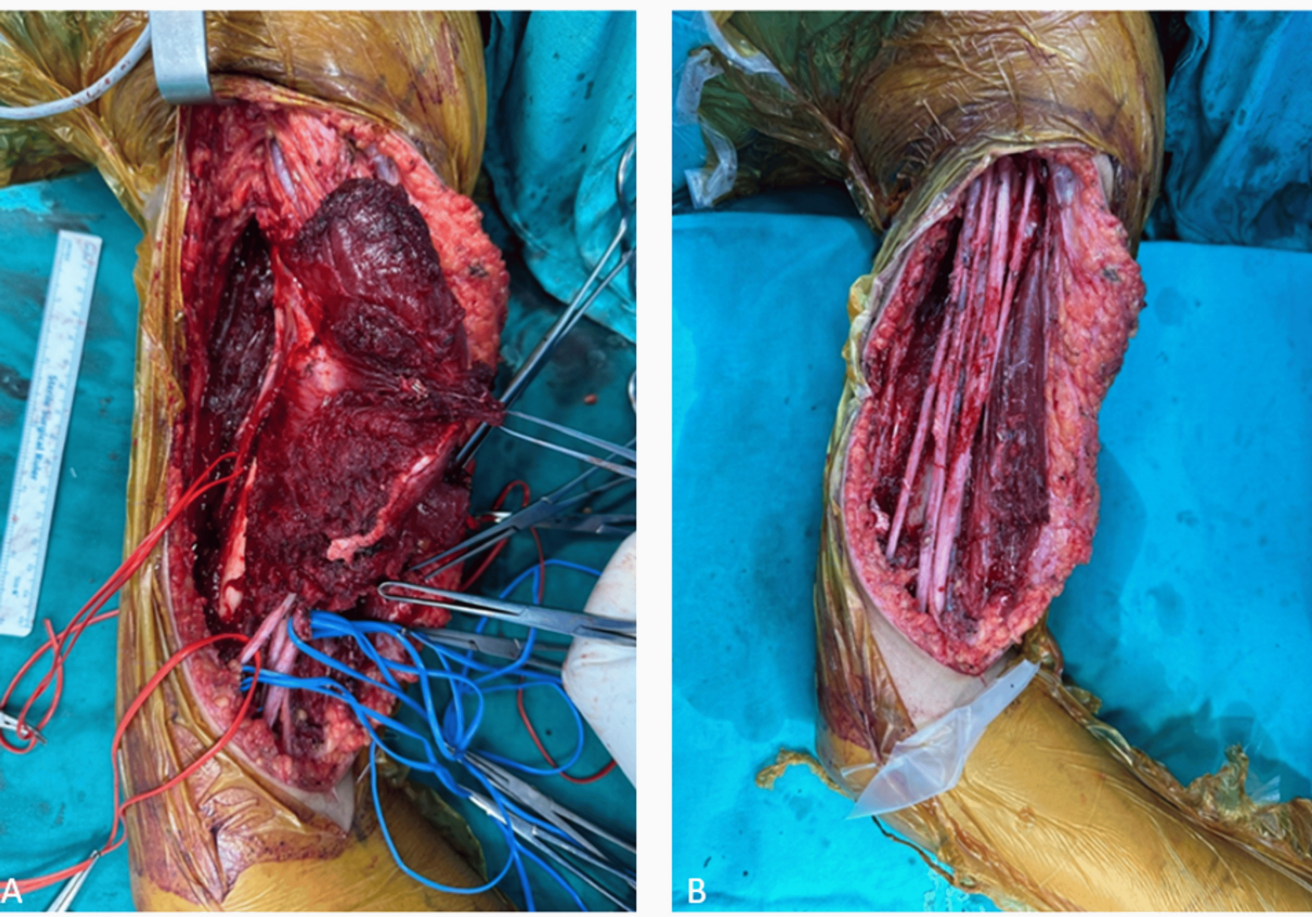
Intraoperative images of the second patient following wide resection surgery: (A) pre-resection and (B) post-resection images.

The excised tumoral tissue was sent for pathological examination, which reported a lesion measuring 72 × 102 × 86 mm (Figures 4, 5).

Following tumor resection and confirmation of hemostasis, the patient's distal circulation was assessed and found to be intact. The remaining muscles and subcutaneous tissues were meticulously sutured, and a hemovac drain was placed to manage postoperative drainage. Postoperatively, the arm was immobilized with a long arm cast and a shoulder-arm sling, maintaining the elbow at 95 degrees of flexion to facilitate proper healing.

Postoperatively, the patient underwent chemotherapy with a regimen including doxorubicin, cyclophosphamide (Endoxan), mesna, and vincristine, in addition to radiotherapy treatments. During a one-year follow-up period, the patient attended regular outpatient clinic visits. No evidence of metastasis or recurrence was detected during postoperative evaluations, indicating a favorable outcome (Table 1).

**Table 1:** Clinical information summary for the two patients.

Category	Case 1	Case 2
Age	60	18
Sex	Female	Female
Location	Interscapular region	Right arm
Size of the lesion	53 x 43 x 25 mm	72 x 86 x 102 mm
Invasion into surrounding soft tissue	Teres major muscle invasion suspected	Biceps brachii muscle, triceps brachii muscle, brachioradialis muscle
Surgery	Wide resection	Wide resection
Pathological diagnosis	Extraskeletal Ewing sarcoma, EWSR-1 (22q12) rearrangement +	Extraskeletal Ewing sarcoma, EWSR-1 (22q12) rearrangement +
Metastasis	None	None
Wound site problem	Clean wound site, discharge after 3 days	Clean wound site, discharge after 6 days
Recurrence	No recurrence	No recurrence
Follow-up period	1.5 years	1.5 years

## Discussion

Ewing sarcoma arising in soft tissues is significantly less common than its osseous counterpart, accounting for approximately 6-7% of primary malignant bone tumors. It predominantly affects males and is the second most common bone tumor in young individuals aged 5-25 years [10]. While it typically occurs in short and flat bones, with the femur and pelvis being the most common sites, our clinic encountered unusual cases involving tumors in atypical locations: the interscapular region in a 60-year-old and the biceps in an 18-year-old patient. These locations contrast with the more frequently reported sites near the spine or retroperitoneum. Despite its rarity, we treated two cases of EES within one year.

Surgery is a cornerstone of EES management, typically combined with chemotherapy and radiotherapy. Standard protocols include four to six cycles of chemotherapy with agents such as doxorubicin (Adriamycin), cyclophosphamide, and vincristine, supplemented by surgical approaches tailored to tumor staging. This multimodal treatment strategy achieves a survival rate of approximately 70%, with more favorable outcomes in pediatric patients. Prognosis is influenced by factors such as tumor size, blood markers, and complete surgical excision. The 10-year survival rate for EES is approximately 55-60%. Both patients in our case report underwent surgical excision of the lesion followed by chemotherapy. Neither experienced wound site complications or recurrence after completing their treatment regimen. The patient with the arm lesion required an extended chemotherapy course due to the lesion's larger size [11,12].

The most common symptoms of EES include pain, swelling, and elevated blood markers. Radiologically, it may display patterns such as an "onion-skin" appearance and periosteal reactions. MRI is the preferred modality for assessing tumor extension, while CT scans and PET imaging aid in detecting metastases to lymph nodes and distant organs. Histologically, EES is composed of small round cells forming rosette-like structures, with intracytoplasmic glycogen highlighted by special stains. This aggressive tumor often metastasizes to the lungs, lymph nodes, skeleton, and brain. In our cases, PET-CT scans identified potential metastases, and MRI evaluated tumor extension [13]. Both patients with interscapular lesions showed no signs of distant metastasis. However, the patient with the arm lesion presented with a suspected area in the proximal right femur during PET-CT scans. Subsequent MRI ruled out metastasis in this region. One year after surgery, whole-body PET and MRI scans confirmed no evidence of recurrence or metastasis in either patient.

After the initial diagnosis of Ewing sarcoma through MRI, excisional biopsy surgery was performed to remove the masses. While a direct excisional biopsy could have been considered based on preliminary MRI findings, current literature recommends an initial biopsy to confirm the diagnosis and determine the necessity for neoadjuvant therapy. Following biopsy confirmation in our two patients, a surgical-wide resection was performed. A study by Takenaka et al. [14] examined treatment outcomes and prognostic factors for EES and osseous Ewing sarcoma in Japanese patients. The results demonstrated improved survival rates for Ewing sarcoma in the last decade, with comparable outcomes between Japanese and Caucasian patients. Interestingly, the study reported a better prognosis for patients with extraskeletal involvement compared to skeletal Ewing sarcoma [14].

Functional assessments and postoperative quality-of-life outcomes are vital in evaluating EES treatment success. In our cases, both patients demonstrated encouraging recoveries. The patient with the interscapular lesion regained full shoulder function within six months, reporting no activity limitations. The patient with the arm lesion required longer rehabilitation due to extensive resection but achieved functional independence and resumed most activities by the one-year follow-up. Despite these positive outcomes, the absence of formal quality-of-life surveys or standardized assessments limits the evaluation of long-term recovery. Future research should include validated tools to provide a more comprehensive understanding of functional and quality-of-life outcomes.

Our study's follow-up period ranged from 1.5 to two years, which is insufficient for assessing long-term outcomes, particularly recurrence or metastasis. Additionally, the study is limited by its small sample size, as only two patients were included. Extending the follow-up to five years and increasing the number of patients would provide more meaningful insights into long-term prognostic trends.

## Conclusions

EES is a rare malignancy that requires a multidisciplinary approach for optimal management. While treatment strategies vary, surgical resection with negative margins remains the cornerstone, often combined with adjuvant chemotherapy to reduce recurrence and improve survival. Early diagnosis and timely intervention are crucial for achieving favorable outcomes.
